# Monthly Summary

**Published:** 1881-09

**Authors:** 


### MONTHLY SUMMARY.

---

"*Fooled by Temperature.*—In his little book on Semeiology, lately published, Dr. J. Milner Fothergill has this passage: Often a rise or fall in the temperature heralds a coming change, of which it may be the first outward sign. On the other hand, the student must know that at times rapid rises of temperature are nervous in origin, are, in fact, "true neurosee." In one case which came under my notice, in a very nervous girl, for months the temperature, when taken, was over 103°. This rise was accompanied by increased rapidity in the respiration and the pulse. Yet she was sinking of inanition, and never approached the typhoid condition which is the consequence of a sustained high temperature, nor gave any indication of persisting fever. Once the temperature, when taken, was 104°, yet she was not at all "feverish;" it was just excitement, and too evanescent to produce any distinct consequences. Further, listen to what Austin Flint says: "The physician is liable to be misled by placing too much reliance on the laws of temperature. They are not infrequently interfered with by complications and accidental events. As an illustration, a young girl had passed through typhoid fever, convalescence being declared, in connection with other symptoms, by the laws of thermometry belonging to the decline of fever or defervescence in this disease. Suddenly hysterical symptoms were manifested, and the temperature rose to 105°. The physician, a man of learning and large experience, was naturally alarmed. In a few hours, however, the temperature declined, and recovery took place without further impediment. The expressive comment made by the physician was: "This is not the first time I have been fooled by temperature!" With regard to the information furnished by the thermometer, as well as other diagnostic symptoms, it is to be borne in mind that there are exceptions to rules which are generally applicable. It is in the female sex that these neurosal disturbances are usually manifested. At the catamenial week of the menstrual cycle, temperature perturbations are common, and a pyrexia, for which there is no apparent cause, may at these times cause unnecessary alarm. *Experte Credo!—Pacific Med. & Surg. Journal.*

---

*An Extraordinary Nephritic Calculus.*—At the recent meeting of the American Medical Association at Richmond, Dr. J. E. Reeves, of Wheeling, presented, from Dr. B. W. Allen, of Wheeling, a report of a case of nephritic calculus weighing 480 grains, which had caused a pyo-nephrosis with a large fluctuating tumor of the hypochondrium. By aspiration eighteen pounds of sero-purulent fluid were evacuated, with temporary relief. The patient died, and on autopsy the left kidney was found to have been converted into a sac, fifteen inches long, twelve inches wide, and six inches deep, containing ten pounds of purulent fluid, together with the above-mentioned calculus. —*Pacific Med. & Surg. Journal.*

*Bromide of Ethyl as an Anæsthetic.*—Dr. R. J. Lewis, in the *Phila. Med. Times*, gives the results of his observations as to the anæsthetic proprieties of this drug. It is characterized by rapidity of action and quick recovery from its effects. It does not influence the circulation to any marked extent, nor is there the same danger from cerebral anæmia and fatal syncope as is seen when chloroform is used. In its effects on the respiration, bromide of ethyl resembles ether more than chloroform, producing only ordinary anæsthetic sleep. Nausea and vomiting are less frequent than with other anæsthetics. Its odor resembles somewhat that of chloroform, although it is less agreeable. It also possesses an advantage over chloroform in being entirely eliminated through the lungs. There is less tendency to struggle and to general excitement, when the bromide is used, than when the other anæsthetics mentioned are employed. Anæsthesia is generally produced by this drug in from two to three minutes, and the quantity necessary to produce this result is from one to eleven drachms.—*Druggists Circular.*

---

*A Death from Ether* has occurred at Jefferson College Hospital, Philadelphia. The patient, a woman, aged twenty-six years, was a patient of Dr. J. R. Levis, who was to operate upon her for fibrous anchylosis of the hip. She had taken ether previously without ill effects, and her viscera were all healthy. She took between two and three ounces of ether, and the operation was safely performed. She did not rally from the anæsthetic, however, and in spite of stimulants, an hour and a half after etherization commenced, she died. Post-mortem revealed nothing abnormal. Shock may have had something to do with the fatal result.—*Med. Record.*

---

*Keep Posted.*—A dentist should not only be a gentleman, but he should be a well versed scholar. Perhaps no profession or occupation demands such an amount of information as that of the dentist. He is daily brought in contact with all kinds and character of mankind, from the common laborer to the most gifted and eloquent men and women, and in order for him to be thoroughly at home at all times, it becomes a necessity to read, and read closely, so that he may always be ready to enter into any discussion or conversation. He must not only be thorough in the literature of his own profession, but he must also be thorough in the literature of the day, so that he may be at perfect ease, and free from embarrassment when any new subject is broached. Patients appreciate this, and dentists will find it not only a paying, but useful investment to surround themselves with popular authors on all subjects. Yours is a profession that requires study, and you must be a thorough student as well as dentist, if you desire to reach the top round in the ladder.—*Dental Headlight.*

---

*Alcohol as a Stimulant or Narcotic.*—Alcohol, says Dr. Wilkes, in the *Monthly Magazine of Pharmacy,* is stated to be a stimulant. If a man is jaded and tired, it affords a temporary support; a little later he is depressed, the stimulant lasting only a short time. It produces dilation of the vessels and warmth of the surface, but at the expense of internal heat. The fact is, alcohol is not a stimulant at all, *but a depressant and a narcotic.* If the name were changed we should get a proper notion of its character, and Dr. Wilkes believes that such a change would tend more than anything else to make people cautious in its imbibition. Alcohol is taken for the same reason as chloral or opium in other countries, and if regarded as a narcotic, the consequences of its use would be better understood. It has a sedative effect, and is therefore useful in severe neuralgia when chloral and opium fail.—*Druggists Circular.*

---

*Drilling Glass.*—The following directions were contributed to *Design and Work* by an optician: First make a saturated solution of camphor in spirits of turpentine; then make a *spear shaped drill* the size of the hole required; heat the drill to a white heat and plunge into mercury, and it will then be *very hard;* sharpen on an oilstone, knock drill in a brad-awl handle, dip the end of drill into the above solution, and work it as if you were working it through wood. It is no use fixing the drill in a drillstock, because the motion all one way won't do. Keep the drill well moistened with the solution, and sharpen it when blunt. A file dipped into solution will file the hole larger, and will not get blunt.

*National Dental Association.*—The second annual meeting of the National Dental Association was held in New York City, August 9th and 10th. President Northrop, of New York, in the chair. There were about thirty members in attendance. Dr. F. M. Odell read a paper on "Lithiasis," and Dr. M. L. Rhein followed with one on "Dental Education." These papers were then discussed by the members generally. On the second day the following officers were elected for the ensuing year: President, John B. Rich, of New York; Vice-Presidents, J. B. Patrick, of Charleston, S. C., J. H. Smith, of New Haven, Conn., D. W. H. Dwinelle, of New York, J. R. Walker, of New Orleans, F. A. Levy, of Orange, N. J.; Assistant Secretary, F. M. Odell; Assistant Treasurer, John Allen, of New York.

Dr. Patrick, of Charleston, read a paper concerning a peculiar case of interstitial growth of a tooth. The paper was then discussed by Drs. Abbott, Walker, Patrick, Buckingham, and Odell. A paper read by Dr. Walker, of New Orleans, on "Tooth Culture," was discussed by Drs. Dwinelle, Allen, Abbott, Patrick, Hunt, Ambler, Winecler, and Swift. The association then adjourned until August, 1881, when it will meet in Washington.

---

*Fatty Tumor of the Tongue.*—At the Charité Hospital, M. Gosselin recently observed a rare form of lingual neoplasm, consisting of a true lipoma. The patient was a man aged forty-eight years, who had first noticed a little button at the tip of his tongue, and to the right, when twenty-three years old. Very gradually the tumor had increased in bulk. Special symptoms or inconvenience were not developed; nevertheless, for the past two years phonation had become somewhat impaired. The neoplasm had attained about the size of a walnut, had a distinct pedicle, and did not appear to extend into and between the lingual muscles. At the surface of this submucous growth some ulceration had taken place. The operation consisted of simple ablation. The microscope confirmed the naked-eye diagnosis, and the mucous membrane covering the lipoma was found to be unaltered.—*Lyon Medical.*

